# Positive Interspecific Relationship between Temporal Occurrence and Abundance in Insects

**DOI:** 10.1371/journal.pone.0018982

**Published:** 2011-04-20

**Authors:** Ricardo A. Scrosati, Ruth D. Patten, Randolph F. Lauff

**Affiliations:** Department of Biology, Saint Francis Xavier University, Antigonish, Nova Scotia, Canada; University of Alberta, Canada

## Abstract

One of the most studied macroecological patterns is the interspecific abundance–occupancy relationship, which relates species distribution and abundance across space. Interspecific relationships between temporal distribution and abundance, however, remain largely unexplored. Using data for a natural assemblage of tabanid flies measured daily during spring and summer in Nova Scotia, we found that temporal occurrence (proportion of sampling dates in which a species occurred in an experimental trap) was positively related to temporal mean abundance (number of individuals collected for a species during the study period divided by the total number of sampling dates). Moreover, two models that often describe spatial abundance–occupancy relationships well, the He–Gaston and negative binomial models, explained a high amount of the variation in our temporal data. As for the spatial abundance–occupancy relationship, the (temporal) aggregation parameter, *k*, emerged as an important component of the hereby named interspecific temporal abundance–occurrence relationship. This may be another case in which a macroecological pattern shows similarities across space and time, and it deserves further research because it may improve our ability to forecast colonization dynamics and biological impacts.

## Introduction

Macroecology investigates the distribution and abundance of organisms at broad scales [1]. One of the most studied macroecological patterns is the interspecific abundance–occupancy relationship. For species assemblages in a region, there is a relationship between the mean local abundance of each species and the proportion of local sites that each species occupies. Studies done on plants and animals have shown that such a relationship is generally positive [2]. The factors that shape the spatial abundance–occupancy relationship are not entirely clear, although likely ones are niche breadth, habitat selection, vital rates, range overlap, body size, and dispersal [2]–[6]. What is certain is that the search for such factors has stimulated research on the links between species distribution and abundance.

Both species distribution and mean abundance can be viewed across space as well as time [1]. Thus, it is also relevant to investigate whether the temporal distribution and mean abundance of species over time may be related. For this purpose, we define temporal occurrence as the proportion of sampling dates in which a species occurs in a given place, and temporal mean abundance as the number of individuals counted in that place throughout the study period divided by the total number of sampling dates. Finding a link between both traits could have important implications for basic and applied ecology. For example, the frequency of visits of mobile species to an area of interest (e.g., a new agriculture field or a restored habitat) could be predicted from knowledge on the abundance of species in similar neighboring environments, which could help to forecast ecological impact or colonization patterns. Conversely, collecting data from simple counts of species occurrence in traps or species sightings in an area might allow one to estimate species abundances in the region, a useful option when traditional methods of abundance estimation are difficult to implement. Interestingly, however, the possible existence of an interspecific temporal abundance–occurrence relationship remains largely unexplored. To investigate this issue, we used an insect assemblage.

Specifically, we asked whether temporal occurrence and temporal mean abundance are related and, if so, whether the relationship is positive. A number of mathematical models have been proposed to describe the spatial abundance–occupancy relationship, among which the He–Gaston model and the negative binomial model usually yield the greatest fit [7]. These models (see Materials and Methods) are based on the degree of spatial aggregation of species, which is a common feature in plant and animal populations [8]. In fact, this predominant trait of natural systems makes the He–Gaston and negative binomial models theoretically more realistic alternatives than the other proposed models, since the latter are based either only on random distribution patterns or on restricted conditions of aggregation that exclude natural variation [7]. Since aggregated patterns of species occurrence may also happen over time, the He–Gaston and negative binomial models emerged as potentially useful tools to describe temporal relationships. Therefore, we tested their utility for our data.

## Materials and Methods

We used data for a species assemblage composed of horse and deer flies (Diptera, Tabanidae) from South Side Harbour, Nova Scotia, Canada (45°40′N, 61°53′W). The daily occurrence and abundance of 31 species (Table 1) were recorded on 37 consecutive days in June–July 2000 (Data S1) using a trap box located in a hay field surrounded by forest vegetation near freshwater marshes. The box design has been described by French and Hagan [9]; it is particularly suitable to detect the abundance of tabanid fly species in the environment. Because most tabanid flies are diurnal [10], the trap was emptied daily at dusk for measurements. The daily samples were frozen within 30 minutes of being collected. Every collected specimen was analyzed under a stereomicroscope and identified using the taxonomic key developed by Teskey [11].

**Table 1:** Number of days in which each species of tabanid fly occurred in the trap (total n = 37 days) and total number of individuals found for each species during the study period.

Species	Number of days	Total number of individuals
*Chrysops aestuans*	3	3
*Chrysops calvus*	6	7
*Chrysops carbonarius*	1	1
*Chrysops cincticornis*	4	4
*Chrysops cuclux*	1	3
*Chrysops excitans*	31	500
*Chrysops frigidus*	3	6
*Chrysops lateralis*	1	1
*Chrysops mitis*	1	1
*Chrysops niger*	17	38
*Chrysops sordidus*	1	4
*Chrysops vittatus*	2	4
*Hybomitra affinis*	6	16
*Hybomitra arpadi*	2	3
*Hybomitra epistates*	32	531
*Hybomitra frontalis*	5	9
*Hybomitra illota*	1	3
*Hybomitra lasiopthalma*	31	3348
*Hybomitra liorinha*	2	3
*Hybomitra longliglossa*	1	3
*Hybomitra lurida*	2	5
*Hybomitra microcephala*	1	3
*Hybomitra nitidifrons nuda*	16	221
*Hybomitra pechumani*	9	15
*Hybomitra trepida*	24	91
*Hybomitra typhus*	12	20
*Hybomitra zonalis*	3	4
*Tabanus marginalis*	1	1
*Tabanus nigrovittatus*	5	19
*Tabanus reinwardtii*	1	1
*Tabanus similis*	27	181

For each species, we determined temporal occurrence as the proportion of sampling dates in which individuals were found in the trap. We determined the temporal mean abundance of each species as the total number of individuals found in the trap during the study period divided by the total number of sampling dates (37). We investigated the relationship between temporal occurrence and temporal mean abundance by evaluating the fit of the data to the He–Gaston and negative binomial models. The He–Gaston equation is:
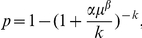
where *p* was originally defined as the spatial occupancy of a species (proportion of surveyed sites occupied by the species), *μ* as the mean local abundance across the surveyed sites, *k* as a spatial aggregation parameter, and *α* and *β* as generic parameters empirically determined on a case-by-case basis [7]. For our study, we considered *p* to be the temporal occurrence of a species, *μ* as the temporal mean abundance of that species, and *k* as a temporal aggregation parameter. The negative binomial model derives from the He–Gaston model simply by considering that both empirical parameters are 1 [7]:
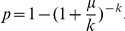



We parameterized both models using nonlinear least-squares regression [12]. We evaluated the degree of model fit by calculating the Pearson correlation coefficient (*r*) between the observed values and model-predicted values of temporal occurrence. In general terms, a perfect fit of a data set to a nonlinear model should produce a perfect correlation (*r* = 1) using all predicted–observed data pairs. We did the analyses using SYSTAT 5.2 for Macintosh.

A number of studies on the spatial abundance–occupancy relationship have calculated the mean local abundance of each species by dividing the total number of organisms found in the region of interest by the number of sampling units in which the species occurred (not by the total number of surveyed units). However, such an alternative measure of mean local abundance produces a number of undesirable artefacts on the abundance–occupancy relationship [13]. Therefore, for our study, we did not consider the equivalent form of temporal mean abundance (that is, temporal mean abundance calculated for each species using only the sampling dates in which individuals occurred in the trap).

As part of our descriptive statistics, we calculated Simpson's evenness index applied to the abundance values for our insect species [14]:
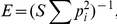
where *S* was the species richness (31) and *p_i_* was the proportional abundance of each species relative to the total number of individuals counted during the study period. Simpson's evenness index ranges between 0 and 1 [14].

## Results

We found 5049 individuals of tabanid flies during the study period (Table 1; Data S1). There was a wide range in the occurrence of species over time, with some appearing only in one date and others almost throughout the entire study period (Table 1). Most species, however, occurred in a limited amount of dates: 8.1±1.8 dates (mean ± SE, n = 31 species). Likewise, species abundance also showed a wide range, with some species contributing with only one individual during the study period and others contributing with many (Table 1). Most individuals, however (96%), belonged to just six species, which yielded a low evenness index for this assemblage (*E* = 0.07).

**Figure 1:**
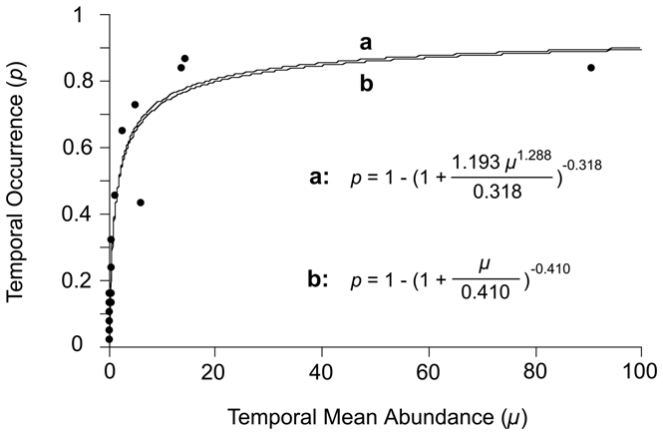
Relationship between the temporal occurrence (*p*) and temporal mean abundance (*μ*) of species of an insect assemblage from Nova Scotia, Canada. The temporal mean abundance was calculated for each species based on all sampling dates (n = 37). The upper line (a) is the He–Gaston model and the lower line (b) is the negative binomial model as parameterized for this data set.

Temporal occurrence and temporal mean abundance were positively related for our insect assemblage. The data showed a high degree of fit to the He–Gaston and negative binomial models. Even by having two empirical parameters (*α* and *β*) in addition to the temporal aggregation parameter (*k*), the He–Gaston model showed only a marginally higher fit (*r* = 0.971, *P*<0.001) than the negative binomial model (*r* = 0.970, *P*<0.001; Fig. 1).

## Discussion

Our study has revealed a positive relationship between temporal occurrence and temporal mean abundance using tabanid flies as a model species assemblage. In addition, two equations that often describe spatial abundance–occupancy relationships well, the He–Gaston and negative binomial models [7], were also found to describe the temporal abundance–occurrence relationship successfully. Since the He–Gaston model yielded only a marginally higher fit than the negative binomial model, the temporal aggregation parameter (*k*) emerges as a key element of the temporal abundance–occurrence relationship. Thus, investigating what determines the timing of occurrence of different species in communities should lead to building functions with appropriate *k* values to predict outcomes under different scenarios.

It is worth noting that a previous study had found a positive correlation between the number of years in which annual plants occurred in an Arizona desert and their overall abundance over time [15]. However, no attempt was made in that study to test the ability of equations developed for the spatial abundance–occupancy relationship to model temporal data, as done here.

The high degree of model fit found for our data set calls for studies on other species assemblages to test for the generality of the temporal abundance–occurrence relationship. It may also be interesting to examine possible links between temporal and spatial patterns and possible effects of community traits such as species richness and evenness or habitat traits such as environmental suitability [16]. The existence of similar patterns across space and time is not infrequent in ecology, although the factors affecting spatial vs. temporal relationships may differ to some extent (for example, biomass–density patterns in crowded plant stands [17], [18]). The aggregation parameter (*k*) appears to be a key component for both the spatial and temporal relationship between species distribution and abundance. Thus, it may also be pertinent to investigate what processes may affect species aggregation in space and time in comparable ways. Overall, we hope that the present study opens the door to long-term research on the fundamental and applied aspects of the interspecific temporal abundance–occurrence relationship.

## Supporting Information

Data S1 Data set in Excel format.
(XLS)
